# Potatoes and Tobacco; Their Introduction into Europe

**Published:** 1883-11

**Authors:** Henry M. Lyman

**Affiliations:** Professor of Physiology and of Diseases of the Nervous System, Rush Medical College, Chicago


### ARTICLE IV.

### POTATOES AND TOBACCO: THEIR INTRODUCTION INTO EUROPE.

By HENRY M. LYMAN, A.M., M.D., Professor of Physiology and of Diseases of the Nervous System, Rush Medical College, Chicago.

Three hundred years ago our ancestors suffered untold misery from scorbutic and gouty disorders, induced through the lack of fresh vegetable provisions during the long, cold winters of Northern Europe.* During the summer months, "asparagrass," spinage, celery, radishes, carrots, cabbages, and turnips were raised in tolerable abundance, and the rich could lay up for the winter good store of dried apples, currants, and gooseberries, with candied cherries, plums, and various nuts; but for the masses nothing better than salted meats, dried fish, old cheese, and much home-brewed beer could be obtained. So late as the reign of Queen Anne it is recorded† that—

"Fruit is brought only to the Tables of the Great, and of a small number even among them. The Desert they never dream of, unless it be a Piece of Cheese."

The same author writes:‡

"The English eat a great deal at dinner; they rest awhile, and to it again, till they have quite stuff'd their Paunch. * * * I have known several people in England that never eat any bread, and universally they eat very little: they nibble a few crumbs, while they chew the meat by whole mouthfuls. * * *

---

* Carpenter's Physiology, 8th ed., pp. 93-4.

† "Social Life in the Reign of Queen Anne," p. 149.

‡ Op. cit., p. 141.

Among the middling sort of people they have ten or twelve sorts of common meats which infallibly take their Turns at their Tables, and two Dishes are their Dinners: a Pudding, for instance, and a Piece of Roast Beef: another time they will have a piece of Boil'd Beef, and then they salt it some Days before hand, and besiege it with five or six Heaps of Cabbage, Carrots, Turnips, or some other Herbs or Roots, well pepper'd and salted, and swimming in Butter. * * * When they have boil'd meat, there is sometimes one of the Company that will have the Broth; this is a kind of Soup, with a little Oatmeal in it, and some Leaves of Thyme or Sage, or other such small Herbs."

At the taverns a gentleman would have "a whet of old Hock to sharpen his appetite for dinner, which consisted of two calves' heads, a couple of geese, and Cheshire cheese."§

No physiologist can be surprised that after such a highly nitrogenized meal "they all fell to drinking wine;" nor is it difficult to comprehend the significance of the child-like delight with which the early English slave traders and buccaneers made trial of the fruits and vegetable of the tropics. Witness Capt. John Hawkins, sailing from Africa with his goodly fleet of ships —the Swallow, the Tiger, the Solomon and the Jesus—all well laden with Samboes and Tangomangoes, captured in Sierra Leone for the South American slave market. The voyage was delayed by calms and tornadoes; the life-ship's crews sweltered under the equatorial sunshine till, in the words * of the quaint old chronicler, they were "put in such fear that many never thought to have reached the Indies without great death of negroes and of themselves; but the Almighty God who never suffereth his elect to perish, sent us on the 16th of February, the ordinary breeze, which is the northeast wind, which never left us till we came to an island of the Cannibals called Dominica." The sailors could there obtain no refreshment; but after beating about the coast for ten or eleven days, they at length found a watering place at Santa Fé, near Cumana. "Near about this place inhabited certain Indians, who the next day after we came thither came down to us, presenting mill and cakes of bread, which they had made of a kind of corn called maize, in bigness of a pease,

§ Op. cit., p. 142.
*Voyages of the Elizabethan Seamen to America. Pp., 21 22.

the ear whereof is much like to a teasel, but a span in length, having thereon a number of grains. Also they brought down to us hens, potatoes, and pines, which we bought for beads, pewter whistles, glasses, knives, and other trifles. These potatoes be the most delicate roots that may be eaten, and do far exceed our parsnips or carrots. Their pines be of the bigness of two fists, the outside whereof is of the making of a pineapple, but it is soft like the rind of a cucumber, and the inside eateth like an apple; but it is more delicious than any sweet apple sugared."

A few weeks later, again reduced to straits for water, our bold captain made the acquaintance of the Indians upon the coast of Florida, where the French were vainly attempting a settlement under the ill-fated Landownière.

"These Indians," writes the chronicler, † "when they travel have a kind of herb dried, who, with a cane and an earthern cup in the end, with fire, and the dried herbs put together, do suck through the cane the smoke thereof, which smoke satisfieth their hunger, and therewith they live four or five days without meat or drink, and this all the Frenchmen used for this purpose; yet do they hold the opinion withal that it causeth water and steam to void from their stomachs."

This was in the year 1565—a year memorable for the introduction of the English to the two American plants which have since revolutionized the dietary of the world.

And yet, notwithstanding the praises of Sir John Hawkins, it was long before the English at home learned to know the value of the new discovery. It was in the year 1586 that the famous voyager, Sir Francis Drake, returning from an expedition to the Spanish Main, carried home to Portsmouth a few roots of the potato.* These were divided between his friends the botanists, Gerard and Clusius. It is probable that the plant had already been carried to Spain, but the grape-loving Spaniards did not highly appreciate the gift. Two years later the brilliant adventurer, Sir Walter Raleigh, a particular friend of Drake, appears upon the bank of the Blackwater, in the south of Ireland, dwelling in a fine old manor-house, long and low, with walls five feet thick, with deep projecting bay windows and porch, orieled

†Op. Cit., p. 46.

*Dic. Encyc. des Sci. Med. Art. Morelle.

closet, high-pointed gables, and great towering chimneys. Here, in the sunny corner, where the town wall of the twelfth century still "bounds the garden of the warden's house, * * * the first Irish potato was planted by him," from the stock brought over by Drake, "and in that garden he gave the tubers to the ancestor of the present Lord Southwell, by whom they were spread throughout the province of Munster."† From Ireland to England, and thence to Germany, was extended the culture of the potato and its companion plant, tobacco. This last herb proven itself the more popular of the two, so that it is recorded‡ that "in Charles the Second's time, in Worcestershire, it was not only usual for the women to join the men in smoking, but that the children were sent to school with pipes in their satchels, and the schoolmaster called a halt in their studies while they all smoked—he teaching the neophytes." Fully two hundred years elapsed, however, before the French had learned the value of the root which had banished scurvy and famine from the homes of the poor. The Erckmann Chatrian "History of a Peasant" very dramatically pictures forth the derisive doubts and the superstitious suspicion with which the simple Alsatian country folk, in the days just preceding the French Revolution, looked for the first time upon the "Hanoverian roots," as they were then called. They would not believe that a *root* could be made to repreduce itself. "Oh, no, you must plant a seed if you would have a crop. Who ever heard of raising carrots and turnips from the root!" And if these things should grow, how could they bring anything but the curse of the heretic Germans by whom they were sent into the holy land of France? Even after it had been demonstrated that potatoes could be raised by Frenchmen, there still remained a widely prevalent belief that eating them would cause the leprosy. It was only when a gentleman, named Parmentier, had prevailed upon the good-natured Louis Sixteenth to eat a potato in full view of the admiring public who daily thronged the royal dining hall, in accordance with the customs of the time, to witness the gorgeous spectacle of their king at his dinner, that the people generally could be persuaded to taste the foreign vegetable.

---

†Nineteenth Century, Nov., 1881, p. 680.
‡Social Life in the Reign of Queen Anne, p. 157.

